# The effect of posterior capsule repair in total hip arthroplasty: a systematic review and meta-analysis

**DOI:** 10.1186/s12891-020-03244-y

**Published:** 2020-04-21

**Authors:** Xiaobo Sun, Xingyang Zhu, Yuqing Zeng, Haitao Zhang, Jianchun Zeng, Wenjun Feng, Jie Li, Yirong Zeng

**Affiliations:** 1 The First Clinical Medical School, Guangzhou University of Chinese Medicine, Jichang Road 12#, District Baiyun, Guangzhou, Guangdong China; 2 Ganzhou Hospital of Traditional Chinese Medicine, Xijin Road 16#, District Zhanggong, Ganzhou, Jiangxi China; 3 Department of Orthopaedics, The First Affiliated Hospital of Guangzhou University of Chinese Medicine, Jichang Road 16#, District Baiyun, Guangzhou, 510405 Guangdong China

**Keywords:** Hip, Replacement, Joint capsule, Repair, Meta-analysis

## Abstract

**Background:**

Prior studies have compared the posterior capsule repair group in primary total hip arthroplasty by posterior approach with the control group without posterior capsule repair suggesting that the posterior capsule repair group had better clinical outcomes. However, it is still a controversy which treatment is more helpful for hip diseases. The purpose of our article is to obtain the postoperative outcomes between the 2 procedures.

**Methods:**

We performed a systematic search by browsing the MEDLINE, EMBASE, Cochrane Library. There is no restriction on the date of publication. Before we submit our manuscript, we have re-searched the literatures again, including the articles which directly compared the postoperative outcomes of the 2 procedures.

**Results:**

A total of 8 comparative studies were included in our meta-analysis. The posterior capsule repair group showed less dislocation rate, higher HHS, and even less postoperative bleeding volume. Meanwhile, there is no significant difference in ROM between 2 groups.

**Conclusion:**

In conclusion, according to current evidences, repairing posterior capsule during primary THA may have better functional outcomes, less dislocation incidence, and less loss of blood.

## Background

Total hip arthroplasty (THA) is a mature and beneficial orthopedic procedure for the treatment of diseases such as osteoarthritis. In the United States, there are more than 300,000 cases of hip fractures each year, and more than 200,000 cases of total hip replacement [1, 2]. And even millions of hip replacements are performed worldwide every year. It can alleviate the pain of patients and improve the function and limb deformity [3, 4]. Primary total hip arthroplasty with posterior approach has the advantages such as less soft tissue injury, shorter operation time, less bleeding volume and faster recovery. At present, posterior approach for total hip arthroplasty is more commonly used than anterior approach [5].

However, the incidence of complications after posterior approach is higher than those after other approaches, especially early postoperative dislocation [6–8]. The complication of dislocation after posterior total hip arthroplasty (PTHA) is very common. Historically, it was reported that the dislocation rate was as high as 0.49 to 4.46% [9, 10].

Papadakis, S.A., et al. suggested that the repair of posterior joint capsule during THA is beneficial to maintain biomechanical stability, promote early functional recovery and reduce the risk of dislocation [11]. Hummel, M.T., et al. thought a larger diameter of the femoral head is helpful to reduce the dislocation of the prosthesis after THA [12]. Nevertheless, Pfirrmann, C.W., et al. have found that even if the posterior joint capsule is strengthened during total hip arthroplasty, most of the posterior soft tissue is not enough to resist the tension of the repair site, which leads to the tear of the repair site [13]. Kao, J.T. and S.T. Woolson also reached a similar conclusion that joint capsule suture is of little significance due to its high failure rate, and there is no significant correlation between postoperative prosthesis dislocation and posterior joint capsule. They believed that repairing the posterior joint capsule does not effectively reduce the risk of prosthesis dislocation after THA, but prolongs the operation time [12, 14].

Although the reconstruction of posterior articular capsule in total hip arthroplasty has been paid more and more attention in recent years, there is a lack of evidence-based medicine to show its superiority. At present, it has not reached consensus on whether the posterior capsule needs repairing during THA. Therefore, in this context, our specific purpose is to quantitatively evaluate whether posterior capsule repair affects the dislocation rate and reduces other complications.

## Methods

### Search strategy

We strictly followed the PRISMA (preferred reporting items for systematic review and meta-analysis) guideline to conduct this analysis [15] according to the Preferred Reporting Items for Systematic Reviews and Meta-Analyses statement. The literature searches for this study were begun after the predefined protocol for this review was registered with PROSPERO by all coauthors and the study protocol was published online at the PROSPERO International Prospective Register of Systematic Reviews. We systematically searched the electronic databases MEDLINE (through PubMed), EMBASE (through OvidSP), and CENTRAL (Cochrane Central Register of Controlled Trials, through the Cochrane Library) for relevant studies without language restrictions applied comparing the main fixation types. The literature search strategy for these 3 databases followed Medical Subject Headings combination with terms (Additional file 1).

### Inclusion and exclusion criteria

Two researchers (Xiaobo Sun and Xingyang Zhu) independently scanned the titles and abstracts of all literatures searched. After excluding the trials which obviously did not meet the inclusion criteria, we read the full text of the literatures that might meet the inclusion criteria to determine whether these literatures completely met the inclusion criteria. In the process of cross-checking, when the data was found to be in doubt, we resolved it by a consensus or through discussion with the third researcher. We also investigated the reference list of each relevant study which was not identified by our original search and determined whether it would be included additionally. Data extraction included demographic data (age, sex, etc.) and available outcome indicators.

All randomized controlled trials (RCTs) and non-randomized controlled trials (nRCTs) directly comparing posterior capsule repair and posterior capsule without repair for patients with primary THA were identified and included from the search strategy. These studies should meet the following inclusion criteria: (1) THA procedure was performed for the first time; and (2) repair of posterior capsule was involved; and (3) the control group was unrepaired posterior capsule group (or capsulectomy group) in the original comparative study; and (4) posterolateral or posterior approach was used; and (5) at least one of the following indexes was reported: operation time, intraoperative bleeding, postoperative drainage, postoperative pain score, time of getting out of bed, postoperative dislocation rate, postoperative hip function Harris score and excellent and good rate evaluation. We also excluded studies that (1) revision of THA was performed; and (2) unclear or incomplete sample data were available; and (3) posterior capsule was not mentioned during procedure; and (4) anterior approach was used; and (5) postoperative effect was inaccurate.

A total of 4624 primary THA cases (4523 patients) were included in this cohort study. 1858 hips in the posterior capsule repair group were compared with 2766 hips in the control group (without posterior capsule repair).

### Study quality assessment

To assess the methodological quality, The nonrandomized studies were assessed using the nine-star Newcastle-Ottawa Scale (NOS), which is a validated tool suitable for assessing the quality of nonrandomized studies [16]. We used the Cochrane risk of bias tool to assess the risk of bias in the RCTs and determine whether biases might have affected the results [17]. Two researchers independently assessed the studies, and disagreements between them were resolved through discussions with a third author or by consensus.

### Data extraction

The data of all included studies were extracted by the first author according to a standardized date collection, and then two other authors independently repeated the process and checked the data extracted by the first author. The date extraction form included the following topics: (1) research features (ie, authors, type of study, journal, year of publication, and country), (2) population information (ie, gender, body mass index [BMI], age, and Primary or non-primary THA), (3) intervention (ie, intraoperative repaired or unrepaired posterior capsule), and (4) outcome index (ie, postoperative dislocation, HHS, etc.). If the necessary results are omitted, we would contact the authors by email or other means to obtain more data if necessary.

### Statistical analysis

In each study, the odds ratio (OR) and relevant 95% confidence interval (CI) were commonly used as the measure of dichotomous variables such as postoperative dislocation rates. Given that the outcome is rare, reported OR was supposed to approximate RR (relative risk) on the basis of Cornfield’s rare disease outcome assumption [18]. The mean difference (MD) or standard mean difference (SMD) was used for continuous variables such as HHS. We used statistical algorithms to estimate the standard deviation for those studies that provided only continuous variables for means and range [19]. Then we included studies where means and standard deviations are available. The I^2^ and Q test were used to evaluate the heterogeneity between studies. The random effects model was in place of the fixed effects model for heterogeneity test, *P* values ≤.1 or I^2^ ≥ 50% [20].

Sensitivity analysis was used to assess the stability of the results (if necessary), and subgroup analysis was conducted to get more specific and detailed results if the data were available. Forest map was used to show the results of each individual study and associated pooled estimates of effect size. Funnel plots was conducted to judge whether there was publication bias for any of the studies. All statistical analyses were conducted using Review Manager (version 5.3.5 for Windows, the Cochrane Collaboration, the Nordic Cochrane Centre, Copenhagen, 2014).

## Results

### Study selection

Seven hundred seventy-eight relevant citations were identified from three databases according to the literature search strategy described earlier. Together with the three additional articles included in other publications, there were a total of 781 studies included. After deleting 105 duplicates, we obtained 676 articles. By reviewing titles and abstracts, 611 irrelevant clinical studies were excluded, and 65 clinical studies remained. By reading the full-text articles, we excluded another 57 articles for the following reasons such as (systematic) reviews, surgery techniques, none-compare groups, cadaver researches, animal researches, TKA patients, no useful outcome date. The remaining 8 articles were deemed appropriately.

**Fig. 1:**
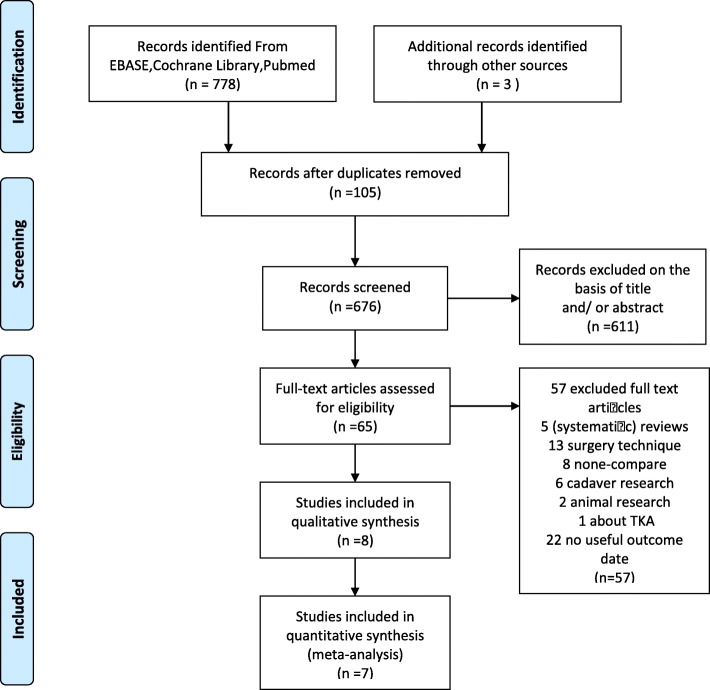
PRISMA flow diagram

There was not combined effect size in one of these 8 studies which were selected. This article was given a descriptive analysis [21]. Finally, 7 publications from 1998 to 2010 were included in our meta-analysis [22, 23, 25–29]. 2 RCT studies were performed for subgroups studies analysis [22, 29]. The detailed literature screening process is shown as PRISMA flow diagram in Fig. 1.

### Study characteristics and quality

Key characteristics of the included studies were provided in Tables 1 and 2. From the 8 citations, 2 RCT [22, 29] studies and 6 nRCT [21, 23, 25–28] studies were identified. 1858 hips in the posterior capsule repair group were compared with 2766 hips in the control group (without posterior capsule repair). All in all, the studies involved about 4624 primary THA cases (4523 patients). Seven studies [22, 23, 25–29] compared dislocation rates and 2 studies [22, 28] compared postoperative HHS between these two groups. Among the 8 studies, 2 studies were from USA [23, 27], 2 from Taiwan, China [22, 26], 1 from Mexico [25], 1 from Korea [28], 1 from Japan [21], 1 from Sweden [29].

**Table 1:** Summary of studies characteristics and patient demographic details for each study

Author	Year of publication	Country	Design	PC/WPC	PTHA/RTHA	Number of THA/Patients	Average age (years)	M/F	Model
Chiu FY et al	2000	Taiwan, China	Prospective randomized study	96/84	PTHA	180/152	G1,51 (25–84) G2,54 (29–90)	G1(59/37) G2(51/33)	Moore
Goldstein WM et al	2001	USA	retrospective study	500/500	PTHA	1000/1000	NR	NR	Gibson
Tsai SJ et al	2008	Taiwan, China	retrospective study	62 (50)/142 (131)	PTHA	204/181	G1,63.32 (35–90) G2,58.55 (24–87)	G1(19/31) G2(63/68)	standard posterolateral approach(a U-shaped capsulotomy)
White Jr. RE et al	2001	Mexico	cohort study	437/1078	PTHA	G1:437/437 G2:1078/1078	NR	NR	standard posterolateral approach
Pellicci PM et al. (the first author)	1998	USA	retrospective study	395/395	PTHA	790/790	NR	NR	posterolateral approach
Pellicci PM et al. (the second author)	1998	USA	retrospective study	124/160	PTHA	284/284	NR	NR	posterolateral approach
Suh KT et al	2004	Korea	cohort study	96/250	PTHA	G1:96/83 G:250/220	G1:53.3 ± 10.8 G2:53.5 ± 10.4	G1(52/31) G2(149/71)	posterolateral approach
Yamaguchi T et al	2003	Japan	cohort study	13/16	PTHA	G1:13/13 G2:16/16	G1:64.3 (52–79) G2:66.8 (50–82)	G1:(2/11) G2:(4/12)	NR
Tarasevicius S et al	2010	sweden	RCT	135/141	PTHA	276/269	G1:69 ± 8 G2:68 ± 9	G1(39/96) G2(49/92)	NR

**Table 2:** Summary of clinic outcomes for each study

Author	Year of publication	Follow-Up	Dislocation (PC/WPC)	Time to Dislocation	Complication (Revision)	Function Score (HHS poiints)
Chiu FY et al	2000	38 M(12-60 M)	0/2	1 (3 weeks Posto) 1 (5 weeks Posto)	NR	G1:Preo49(24–73)Posto93(86–96) G2:Preo45(24–70)Posto91(86–95)
Goldstein WM et al	2001	1y	0.6%(3)/2.8%(14)	NR	G1:3Anteriordislocation,2recrudesce,2revision success; G2:13Posterior dislocation,1Anterior dislocation,13recrudesce,7 revision	NR
Tsai SJ et al	2008	> 12 M	0/6.38%(9)	< 6 M	NR	NR
White Jr. RE et al	2001	> 6 M	G1:3/437,0.7%; G2:52/1078,4.8%	< 6 M	G1:4/437,0.9%, Avulsion fracture of the greater trochanter,< 6 W.	NR
Pellicci PM et al. (the first author)	1998	12 M	0 (0%)/16 (4%)	NR	NR	NR
Pellicci PM et al. (the second author)	1998	6 M	1 (0.8%)/10 (6.2%)	NR	NR	NR
Suh KT et al	2004	1y	1 (1%)/16 (6.4%)	NR	NR	G1:95.2 ± 3.3 G2:93.9 ± 5.5 (1 year postoperative)
Yamaguchi T et al	2003	2 years (range, 1–7.3 years)	NR	NR	NR	NR
Tarasevicius S et al	2010	1y	3 (2%)/7 (5%)	NR	2 sciatic nerve palsies	NR

*PC* Posterior Capsulorrhaphy, *WPC* Without Posterior Capsulorrhaphy, *PTHA* Primary Total Hip Arthroplasty, *RTHA* Revision of Total Hip Arthroplasty, *M* Months, *y* Years, *G* Groups, *G1* PC Group, *G2* WPC Group, *Preo* Preoperation, *Posto* Postoperation, *HHS* Harris Hip Score, *NC* Not clear

The average follow-up time for reported dislocation rates ranged from 6 months to 60 months. The maximum period was 60 months [22], and the minimum period was 6 months [27]. The time of dislocation ranged from at least 1 week to 6 months after surgery. Unfortunately, we did not find specific dislocation time in 4 studies. We contacted the authors, but failed. Mean age ranged from 51 [22] years to 69 (SD 8) [29] years. All the studies included were primary THA, and were divided into two groups. A 28-mm diameter femoral was used in 3 studies [25, 28, 29]. The study characteristics, clinical outcomes, and patient demographic details for the 8 studies are shown in Tables 1 and 2. The methodological quality of involved studies ranged from seven to eight. Table 3 shows a moderately severe risk bias in the included studies.

**Table 3:** Risk-of-bias assessment for the studies included in the meta-analysis (NOS)

(nRCT) Study = 6	Item 1	Item 2	Item 3	Item 4	Item 5A	Item 5B	Item 6	Item 7	Item 8	Score
Goldstein WM et al. (2001) [23]	*	*			*	*	*	*	*	7
Tsai SJ et al. (2008) [24]	*	*		*	*	*	*	*	*	8
White Jr. RE (2001) [25]	*	*	*	*	*		*	*	*	8
Pellicci PM et al. (1998) [27]	*	*		*		*	*	*	*	7
Suh KT et al (2004) [28]	*	*	*	*	*	*	*	*	*	8
Yamaguchi T et al. (2003) [21]	*		*	*	*	*	*	*	*	8
Methodological Assessment According to Six Domains of Potential Biases (Cochrane Risk of Bias Tool)										
RCT Study = 2	Random Sequence Generation	Allocation Concealment	Blinding of Participants and Personnel	Blinding of Outcome Assessment		Incomplete Outcome Data	SelectiveReporting	Other Bias		Overall Bias
Chiu FY et al. (2000) [22]	Low	Low	Unclear	Unclear		Low	Low	Unclear		Low
Tarasevicius S et al. (2010) [29]	Low	Low	Low	Low		High	High	Unclear		High

*NOS* Newcastle - Ottawa Quality Assessment Scale, *RCT* Randomized controlled trial, *nRCT* Nonrandomized controlled trial

### Postoperative dislocation rate

**Fig. 2:**
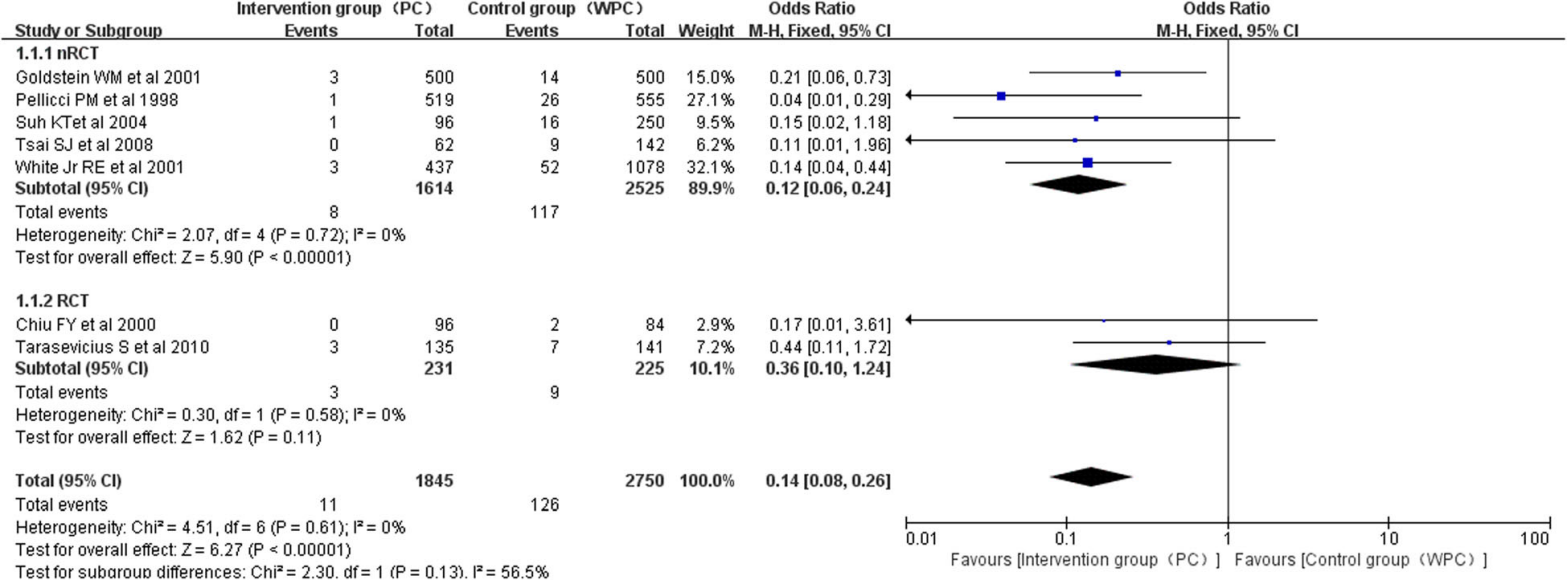
Comparison of the incidence of hip dislocation after primary THA

**Fig. 3:**
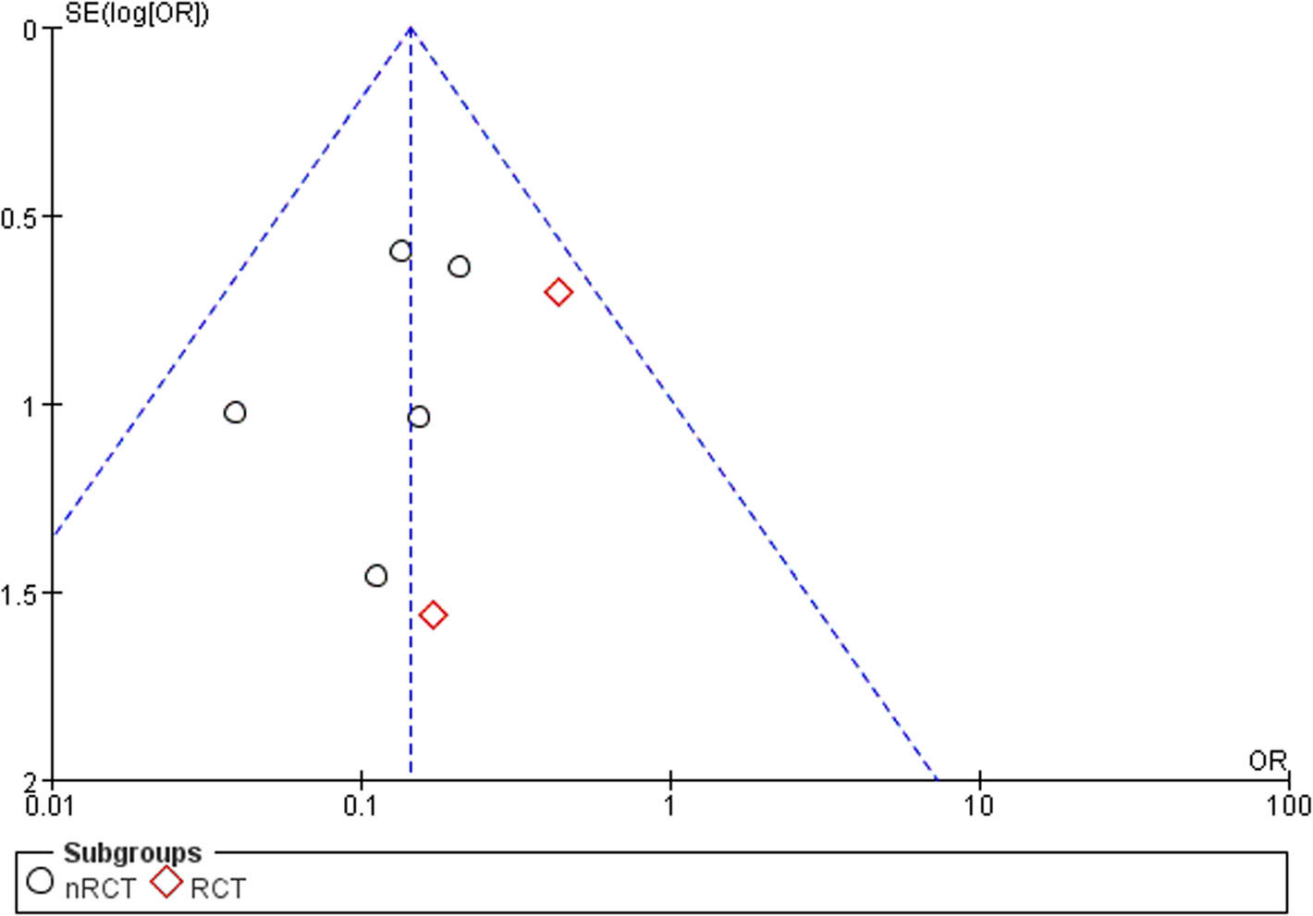
Funnel plots for RCT and nRCTs reporting the dislocation rate

Seven studies [22, 23, 25–29] assessed the incidence of dislocation in a total of 4595 cases. We divided the studies into two subgroups (an RCT subgroup and an nRCT subgroup) for the subgroup analysis. Seven articles clearly reported the rate of postoperative dislocation after primary THA. Heterogeneity test (I^2^ = 0%) indicated that there is statistical homogeneity among the studies, so the fixed effect model was used to analyze. The results of Meta analysis showed that there was a significant difference in the incidence of postoperative dislocation between the repaired capsule group and the unrepaired capsule group (OR 0.14, 95% CI 0.08–0.26, *P* = .61, I^2^ = 0%; Fig. 2). According to the subgroup analysis of the included studies in the literature, the results of the combined analysis of the five nRCT also showed that there was a significant difference between the five groups (OR 0.12, 95% CI 0.06–0.24, *P* = .72, I^2^ = 0%; Fig. 2). The results indicated that the repair of posterior capsule is helpful to reduce the incidence of hip dislocation after primary THA (Table 4). It is of great physiological significance to repair the posterior capsule during total hip arthroplasty. Funnel plots illustrated the meta-analysis of the dislocation rate had no obvious publication bias (Fig. 3).

**Table 4:** Dislocation rate for intervention group (PC) and control group (WPC)

Study	Intervention group (PC)		Control group (WPC)	
	Dislocation	Total	Dislocation	Total
Chiu FY et al. 2000 [22]	0	96	2	84
Goldstein WM et al. 2001 [23]	3	500	14	500
Pellicci PM et al. 1998 [27]	1	519	26	555
Suh KT et al 2004 [28]	1	96	16	250
Tarasevicius S et al. 2010 [29]	3	135	7	141
Tsai SJ et al. 2008	0	62	9	142
White Jr. RE et al. 2001 [25]	3	437	52	1078

### Harris hip score (HHS)

**Fig. 4:**
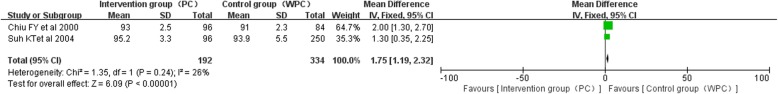
Comparison of the HHS score after primary THA

**Fig. 5:**
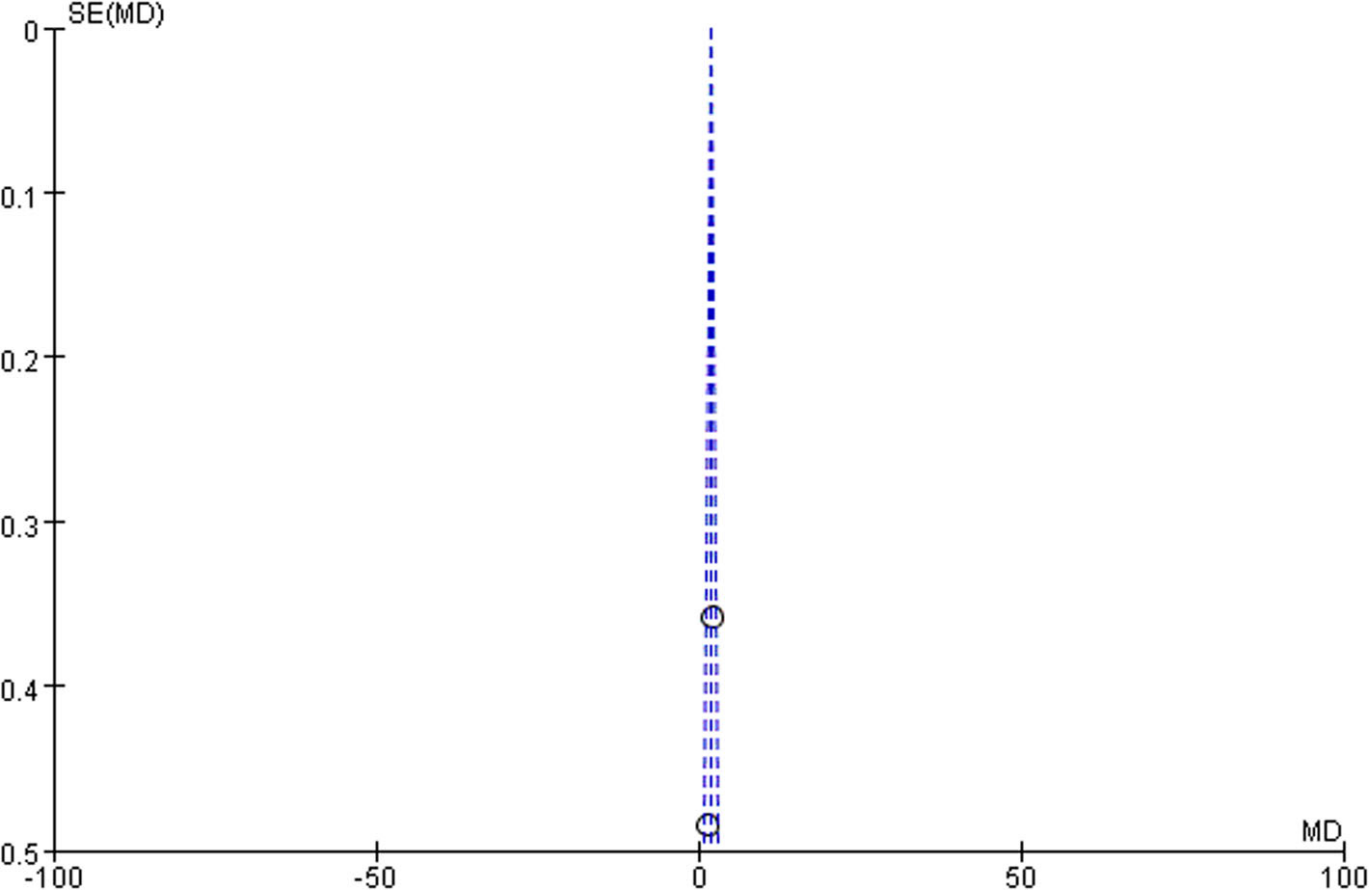
Funnel plots for the studies reporting the HHS score

Two studies [22, 28] used the Harris Hip Score (HHS), and the results showed significant statistical difference between the 2 groups (MD 1.75, 95% CI 1.19 to 2.32, *P* = .24, I^2^ = 26%; Fig. 4). According to the heterogeneity test of the two articles in this study (*P* = .24, I^2^ = 26%), it was suggested that there was no heterogeneity among the selected literatures in this study, so we chose the fixed effect model for meta analysis. In order to ensure the accuracy and stability of the study, we continued to carry out sensitivity analysis. The sensitivity analysis of the two literatures showed that none of them interfered with results of meta analysis, which meant this study had good stability. The MD values of the two studies were 1.75, 95% confidence interval was 1.19 ± 2.32. *P* value (*p* < 0.05) indicated the statistical significant difference between two groups. The results showed that the posterior capsule repair would significantly increase the HHS score after primary THA. The pooled data was showed in the following forest plot (Fig. 4). In this study, funnel plot was drawn to investigate whether publication bias exists. The symmetry of funnel plot means that there is no publication bias. The funnel plot of this study was showed in Fig. 5.

### Complications

Since the effect size of postoperative complications in three original studies [23, 25, 29] was not combined, the pooled data of these complications were unable to be obtained. These postoperative complications included the revision for postoperative dislocation, avulsion fracture of the greater trochanter, sciatic nerve palsies. Goldstein, W.M., et al. reported two cases of revision for dislocation in the capsule repair group and seven cases of revision for dislocation in the group without capsule repair [23]. White Jr., R.E., et al. found that four cases (0.9%) of avulsion fracture of the greater trochanter in the Posterior capsule repair group occurred less than 6 weeks after surgery [25]. Tarasevicius, S., O. Robertsson, and H. Wingstrand demonstrated that 2 sciatic nerve palsies in the repair group were directly relevant to posterior suture [29].

### Range of motion

Three studies [21, 25, 28] assessed range of motion (ROM) of patients. But the pooled data was not obtained because the items of evaluation were different. White Jr., R.E., et al. came to a conclusion that average reduction of the passive internal rotation was 50%, and minimum of the passive internal rotation was 25% in the capsule repair group [25]. Suh, K.T., B.G. Park, and Y.J. Choi reported that the ROM was not affected by posterior capsule repair. There was no statistically significant difference between the two groups [28]. Yamaguchi, T., et al. suggested there was no significant difference in ROM between the two groups before and after surgery [21].

## Discussion

### Main results

In any surgical decision, the safety of patients is always important. Considering the uncertainty and controversy in relation to the influence of posterior capsule repair on the incidence of hip dislocation following primary THA, we sought to evaluate the body of evidence linking posterior capsule repair with the risk of hip dislocation following primary THA carrying out a comprehensive systematic review of observational studies and RCTs. This meta-analysis included 8 studies (2 RCTs and 6 nRCTs) that analyzed 4523 patients (4624 hips) and directly compared the clinical effectiveness of the posterior capsule repair group and the control group without posterior capsule repair. The pooled data indicated significant differences between the 2 groups in terms of dislocation rates, HSS, and so on. The approach of posterior capsule repair in total hip arthroplasty is superior to the control group without posterior capsule repair on the basis of the present evidence base. The risk of hip dislocation was higher in the control group without capsule repair, while the complication rate was lower in the posterior capsule repair group. However, those differences might be caused by the significant between-article heterogeneity which was able to affect accuracy of the outcomes in meta-analysis. The influence of heterogeneity might be reduced by using Random effects model, subgroup analysis, or sensitivity analysis to pool data. However, it does not eliminate it. The current evidence base—with a good number of methodological inadequacies such as the limited usage of power calculations and poor or absent blinding of both patients and assessors—can cause assessor bias. The evidence base was also guilty of documenting methods of recruitment, thus permitting allocation or recruitment bias. In relation to these factors, the presented evidence base, being substantial in size, may be questioned regarding to its quality.

At present, the primary THA has become a routine treatment for hip diseases such as osteonecrosis of the femoral head (ONFH), developmental dysplasia of hips (DDH), femoral neck fractures, osteoarthritis of the hip, ankylosed hip, and so on. Hip replacement operations restore patients’ function earlier, and reduce bedridden complications and mortality [30]. However, with the large-scale popularization of THA surgery, there are more and more complications after THA. The complications of hip replacement can’t be ignored, in which postoperative dislocation is catastrophic [31]. The complication of postoperative hip dislocation is second only to aseptic loosening. Currently, the posterior approach is commonly used in total hip arthroplasty, which has the advantages of a short operative time, less bleeding and a shorter hospital stay. However, its disadvantage is also the higher rate of postoperative dislocation [32].

The reasons for postoperative hip dislocation include patient-related factors, such as sex, hip dysplasia, previous hip surgery, patient compliance, and neurologic compromise [33–36]. Currently, it is believed that there are three major reasons for the high incidence of posterior dislocation of the posterolateral incision. Firstly, the Short external rotation muscle and the joint capsule are cut off in the posterior approach without careful repair when the incision is closed, resulting in the imbalance of the structural stability of the posterior soft tissue. Secondly, due to insufficient exposure of the surrounding soft tissue in the posterior approach, the prosthesis is easily pushed back when placing the acetabular cup, which changes the forward angle of the acetabular cup. That is to say, the incorrect position of the prosthesis affects the postoperative stability of the hip joint to a great extent. Thirdly, the unskilled surgical technique of the operator will also increase the rate of postoperative dislocation.

Some scholars investigated the reasons for dislocation considering prosthesis. McCollum, D.E. and W.J. Gray thought that the misplacement of acetabular components is considered to be an important cause of postoperative dislocation of the hip joint as well as Coventry, M.B., et al. did [9, 37]. Etienne, A., Z. Cupic, and J. Charnley indicated that the dislocation rate after hip arthroplasty is related to the design of prosthesis [38]. Cobb, T.K., B.F. Morrey, and D.M. Ilstrup suggested that an elevated liner can improve the stability of the hip joint after THA, particularly in hips which are at greater risk for the prosthesis dislocation [39]. Sierra, R.J., et al. suggested using a 32-mm head in association with posterior joint capsular repair should reduce the rate of dislocation [40]. Hummel, M.T., et al. recommended that larger femoral head size and joint capsule tissue repair can increase the stability of the hip joint [12]. McCollum, D.E. and W.J. Gray provided a safe angle for acetabular cup placement to prevent dislocation [9]. Larger femoral head sizes can reduce the incidence of dislocation [41–43]. Other scholars linked the stability of the posterior capsule to the posterior dislocation after THA. Clayton, M.L. and R.G. Thirupathi thought that the brace treatment after total hip arthroplasty can improve the stability and reduce the rate of recurrent dislocations [44]. Wu, H.M. and A.Y. reached a decision that posterior capsule repair can reduce the rate of early dislocation after primary THA (within the first 6 months after surgery) [25]. Two senior authors used the method of posterior soft tissue enhancement to prove that posterior capsule repair can prevent postoperative posterior dislocation of the total hip arthroplasty through a posterior approach [27]. Dixon, M.C., et al. reported a simple capsulorrhaphy following a posterior approach which minimized the incidence of postoperative dislocation [45]. Hedley et al. described a method of preserving the posterior capsule for primary hip replacement, with only 2 out of 259 cases of dislocation [46]. Pierchon, F., et al. demonstrated that soft tissue imbalance was the major cause of dislocation by CT scan [47]. Stahelin, t. et al. confirmed through experiments that the posterior capsule of the hip joint needs higher torque to repair dislocation [48]. However, some scholars thought that the technique of posterior capsule suture often failed. Stahelin, T., et al., American scholars, placed markers which could not pass through X-rays during the operation, and observed the distance between the two markers through postoperative X-ray plain films. As a result, even though the posterior capsule was repaired in total hip arthroplasty, and most of them were not strong enough to resist the tension of the repair during the healing position, eventually the repair failed. Therefore, in terms of posterior structure suture techniques, it is necessary for us to further explore better techniques to improve the curative effect [49].

### Limitations and strengths

The limitations of this study are as follows. Firstly, most of the included studies are observational studies (case-controlled studies), in which the reliability of the conclusion needs to be confirmed, because they are more likely to be biased and the number of included literatures is relatively small. Secondly, there is diversity in the design of the prosthesis such as the size of the femoral head and so on, or it has not been reported in detail, so the heterogeneity is increasing among included studies. 28 mm ceramic heads were used in 3 studies, but it is not clear in other articles. We tried to contact the authors, but failed. So we cannot perform a subgroup analysis to see if the dislocations rates were different with studies reporting larger head sizes (36 mm and more) versus smaller head sizes. Thirdly, gender, age and specific diseases in the included studies are significantly different. Thus, it also becomes a possible source of heterogeneity. Fourthly, some studies have been omitted because of the search strategy we performed. To overcome this limitation, we have consulted researched synonyms, optimized the search strategy, and conducted an extensive search. Fifthly, it was not clear whether or not TXA was used in the eight studies included. We also tried to contact the authors, but failed. So this may be a big confounding effect. Sixthly, performing a true RCT is difficult. Only 2 of 8 studies were RCTs, in which it was very difficult to realize the blind method. In most of the studies, the method of operation depended on the patient and the doctor; thus, it was difficult to ensure the baseline balance between the 2 groups. Kwon, M.S., et al. performed a meta-analysis to compare dislocation rates with and without soft tissue repair after THA. However, the posterior capsule repair had not been specifically described in this systematic literature review [10]. Compared with the above-mentioned meta-analysis, we included some new clinical researches, and our outcomes are more up to date. We performed this study in line with the PRISMA statement, while some authors studied in accordance with the Chinese Cochrane Center. To strengthen our meta-analysis, we need further prospective randomized trials investigating dislocation incidence and other clinical parameters.

## Conclusions

In summary, our meta-analysis has shown that posterior joint capsule repair can increase joint stability, reduce hip joint dislocation after THA by posterior approach, increase postoperative HHS, and even reduce the amount of bleeding. We look forward to achieving detailed studies with higher quality in the future. In order to resolve this problem, RCTs on the basis of further systematic and comprehensive evaluation are needed to confirm these findings. It is proposed to provide more reliable objective evidence.

## Supplementary information

**Additional file 1.** Pubmed search stragety.

## Data Availability

The datasets used and/or analyzed during the current study are not publicly available due to feasibility but are available from the corresponding author on reasonable request.

## Abbreviations

NOS: Newcastle - Ottawa Quality Assessment Scale
RCT: Randomized controlled trial
nRCT: Nonrandomized controlled trial
