# Supplementary material for: The effect of posterior capsule repair in total hip arthroplasty: a systematic review and meta-analysis
Source: BMC Musculoskelet Disord. 2020 Apr 21;21:263. doi: 10.1186/s12891-020-03244-y (PMC7175585; doi:10.1186/s12891-020-03244-y)
Supplement: Supplementary file 1 — Additional file 1. Pubmed search stragety. [file 12891_2020_3244_MOESM1_ESM.docx]

**pubmed search stragety**

**((("Arthroplasty, Replacement, Hip"[Mesh]) OR (Arthroplasty, Replacement, Hip[Title/Abstract]) OR Arthroplasties, Replacement, Hip[Title/Abstract]) OR Arthroplasty, Hip Replacement[Title/Abstract]) OR Hip Prosthesis Implantation[Title/Abstract]) OR Hip Prosthesis Implantations[Title/Abstract]) OR Implantation, Hip Prosthesis[Title/Abstract]) OR Implantations, Hip Prosthesis[Title/Abstract]) OR Prosthesis Implantation, Hip[Title/Abstract]) OR Prosthesis Implantations, Hip[Title/Abstract]) OR Hip Replacement Arthroplasty[Title/Abstract]) OR Replacement Arthroplasties, Hip[Title/Abstract]) OR Replacement Arthroplasty, Hip[Title/Abstract]) OR Arthroplasties, Hip Replacement[Title/Abstract]) OR Hip Replacement Arthroplasties[Title/Abstract]) OR Hip Replacement, Total[Title/Abstract]) OR Replacement, Total Hip[Title/Abstract]) OR Hip Replacements, Total[Title/Abstract]) OR Replacements, Total Hip[Title/Abstract]) OR Total Hip Replacements[Title/Abstract]) OR Total Hip Replacement[Title/Abstract]) OR THA[Title/Abstract]) OR THR[Title/Abstract])))) AND (("Joint Capsule"[Mesh]) OR (((((((((((((Joint Capsule[Title/Abstract]) OR Capsule, Joint[Title/Abstract]) OR Capsules, Joint[Title/Abstract]) OR Joint Capsules[Title/Abstract]) OR Capsula Articularis[Title/Abstract]) OR Synovial Capsule[Title/Abstract]) OR Capsule, Synovial[Title/Abstract]) OR Capsules, Synovial[Title/Abstract]) OR Synovial Capsules[Title/Abstract]) OR Articular Capsule[Title/Abstract]) OR Articular Capsules[Title/Abstract]) OR Capsule, Articular[Title/Abstract]) OR Capsules, Articular[Title/Abstract]))**
